# Low-Temperature Formation and Photophysics of Phloroglucinol-Derived Carbonaceous Materials Under Air and Vacuum

**DOI:** 10.3390/ijms27156632

**Published:** 2026-07-25

**Authors:** Chiara Olla, Luigi Stagi, Daniele Chiriu, Carlo Maria Carbonaro

**Affiliations:** 1 Department of Physics, University of Cagliari, Cittadella Universitaria di Monserrato, 09042 Monserrato, Italy; chiara.olla@dsf.unica.it (C.O.); daniele.chiriu@dsf.unica.it (D.C.); 2 Institut Néel, CNRS, 38042 Grenoble, France; luigi.stagi@neel.cnrs.fr

**Keywords:** phloroglucinol, carbonaceous materials, low-temperature carbonization, reaction atmosphere, photoluminescence, transient absorption spectroscopy, Raman spectroscopy, density functional theory

## Abstract

Phloroglucinol is an oxygen-rich aromatic precursor whose thermal evolution can yield emissive carbonaceous materials with excitation-dependent photoluminescence. However, the influence of the reaction atmosphere on its low-temperature transformation remains insufficiently understood. In this work, phloroglucinol was thermally treated at 200 °C under air or static vacuum for 5 and 10 h. The resulting materials were investigated by electron microscopy, energy-dispersive X-ray spectroscopy, Raman spectroscopy, steady-state and time-resolved photoluminescence, transient absorption spectroscopy, and density functional theory calculations. The reaction atmosphere mainly affected the early stages of structural evolution. Air-treated samples formed irregular networks of filamentous substructures, whereas vacuum-treated samples displayed more compact quasi-spherical aggregates with a fibrous internal organization. Raman spectra indicated the progressive transformation of crystalline phloroglucinol into a disordered carbonaceous network containing small sp^2^-rich domains. Optical measurements revealed violet/deep-blue and cyan emissive centers whose relative contributions depended on atmosphere and treatment time. Transient absorption supported a multi-center photophysical picture involving ultrafast relaxation, intermediate trapping or interconversion, and longer-lived decay. Calculations on representative molecular motifs identified furan-containing conjugated domains as plausible candidates for the violet/deep-blue centers and compact triangular phloroglucinol-derived structures for the cyan center. Overall, oxygen availability and treatment duration modulate the morphology and emissive-center distribution of phloroglucinol-derived carbonaceous materials.

## 1. Introduction

Carbonaceous materials obtained from molecular precursors have attracted considerable interest because their structural organization, surface chemistry, and optical response can be modulated through relatively simple synthetic procedures [1,2,3,4,5]. Among the available preparation methods, direct thermal treatment offers several advantages, including limited use of reagents, straightforward processing, and the possibility of controlling the degree of condensation through temperature, treatment time, and reaction atmosphere [6,7,8]. At relatively low temperatures, however, the conversion of organic precursors generally produces chemically and structurally heterogeneous materials containing residual molecular units, oxygen-containing groups, and small conjugated domains [9,10]. Understanding how the synthesis conditions direct this intermediate stage of carbonization is therefore essential for establishing reliable relationships between processing, structure, and functional properties [11,12,13].

Phloroglucinol (PG), or 1,3,5-trihydroxybenzene (C_6_H_6_O_3_), is an oxygen-rich aromatic molecule that represents a particularly interesting precursor for the preparation of carbonaceous materials. Its three-fold symmetric structure combines a thermally stable aromatic ring with three hydroxyl groups that can participate in dehydration, etherification, condensation, and cyclization reactions [14,15]. As a consequence, thermal treatment of PG can generate a range of interconnected aromatic, ether-like, and furan-containing motifs while retaining a substantial oxygen content. These structural features can favor dispersibility in polar solvents and give rise to complex electronic states relevant to functional carbonaceous materials [16,17,18,19,20,21,22,23].

PG has also been used as a molecular precursor for emissive carbon-based systems such as carbon dots [24,25,26]. In particular, hydrothermal condensation has been reported to produce triangular carbon dots with narrow emission bands and potential applications in light-emitting devices [27,28]. These studies demonstrate that the symmetry and reactivity of PG can promote the formation of compact conjugated structures with distinctive optical properties. Nevertheless, materials produced through direct thermal treatment differ substantially from well-defined hydrothermal nanodots, because low-temperature heating generally yields heterogeneous carbonaceous networks rather than uniform nanoscale particles [22,23,24,29,30].

Previous investigations of the thermal behavior of PG have shown that intermolecular cross-linking and condensation can occur with relatively limited mass loss up to approximately 350 °C. Unlike high-temperature pyrolysis routes aimed at maximizing structural ordering and electronic conductivity [31,32], low-temperature thermal treatments provide access to intermediate carbonization states in which molecular fragments and oxygen-rich motifs are still preserved. These intermediate structures are particularly valuable for investigating the emergence of localized emissive centers and their relationship with the evolving carbonaceous framework. Ether formation, dehydration, and the development of furan-containing structures have been proposed as important steps in the progressive stabilization of the material [9,10,33,34]. The complexity of hydration and de-hydration processes of PG, as reported through TGA and DSC analysis, also largely affects PG thermolysis [15]. At the same time, residual hydroxyl groups and oxygen-containing linkages limit the extent of carbonization and preserve a partially molecular character. The resulting products therefore occupy an intermediate regime between the crystalline precursor and a fully developed carbon network.

As already shown for nano and micro-structured carbonaceous materials, the thermal evolution of PG is also strongly dependent on the reaction environment [35,36,37,38]. Studies conducted under inert gas have indicated that reducing oxygen availability can alter the balance among condensation, dehydration, and cyclization pathways, with consequences for the resulting structural organization and solubility. Conversely, treatment in the presence of oxygen may introduce additional oxidative pathways and promote heterogeneous cross-linking [9,10,34]. Despite these indications, the specific role of the surrounding atmosphere in controlling the morphology, structural evolution, and photophysical response of thermally treated PG remains insufficiently understood.

In particular, a systematic comparison between air and strongly oxygen-depleted conditions at the same low treatment temperature is still lacking. Such a comparison is important because atmosphere-dependent effects may be most pronounced during the early stages of thermal evolution, before prolonged heating drives the material toward a more extensively condensed carbonaceous framework. Moreover, while steady-state photoluminescence can reveal the coexistence of different emissive contributions, time-resolved measurements are required to determine whether changes in synthesis conditions modify the intrinsic excited-state dynamics or mainly alter the relative population of the optically active motifs [25,27,39].

In this work, phloroglucinol was thermally treated at 200 °C under air and static vacuum for 5 and 10 h. Additional experiments at 250 and 300 °C were performed to assess the effect of increasing temperature on the resulting morphology. The materials obtained at 200 °C were investigated by scanning electron microscopy, energy-dispersive X-ray spectroscopy, Raman spectroscopy, UV–visible absorption, excitation–emission mapping, time-resolved photoluminescence, and transient absorption spectroscopy. Density functional theory calculations were also carried out on representative PG-derived oligomeric, furan-containing, mixed, and compact triangular motifs to provide a molecular-level framework for interpreting the observed excitation channels.

By correlating morphology, structural signatures, steady-state optical response, and excited-state dynamics, this study examines how oxygen availability and treatment duration influence the low-temperature evolution of PG-derived carbonaceous materials. The results show that the reaction atmosphere primarily affects the mesoscale organization and the relative distribution of emissive centers, whereas prolonged treatment promotes partial convergence toward a similar disordered carbonaceous framework. The combined experimental and computational analysis supports a photophysical model involving multiple structurally related conjugated motifs, with furan-containing domains contributing to the violet/deep-blue emission and compact triangular PG-derived structures representing plausible candidates for the cyan-emitting center.

## 2. Results

### 2.1. Morphological and Compositional Characterization

To correlate the synthesis conditions with the morphology, structure, and optical properties of the obtained phloroglucinol-derived materials, a multi-technique characterization approach was adopted, combining microscopy, elemental analysis, vibrational and optical spectroscopy, ultrafast spectroscopy, and computational modeling. To simplify the discussion, the samples are labeled as PG200A5, PG200V5, PG200A10, and PG200V10, where PG denotes phloroglucinol, 200 indicates the treatment temperature in °C, A or V indicates the treatment atmosphere, air or static vacuum, respectively, and the final number indicates the treatment duration in hours.

**Figure 1:**
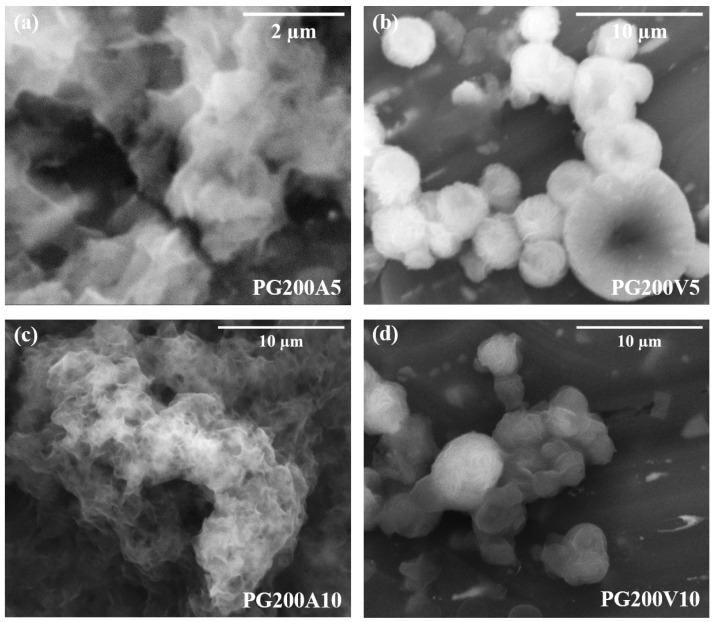
SEM micrographs of phloroglucinol-derived materials obtained after thermal treatment at 200 °C under different atmospheres and reaction times: (**a**) PG200A5, treated in air for 5 h; (**b**) PG200V5, treated under static vacuum for 5 h; (**c**) PG200A10, treated in air for 10 h; and (**d**) PG200V10, treated under static vacuum for 10 h.

The synthesized samples appear as micrometer-scale aggregates whose morphology depends on the treatment atmosphere and duration, as shown in Figure 1. The PG200A5 sample (Figure 1a) consists of irregular aggregates, typically on the order of 1–2 μm, formed by the assembly of smaller and poorly defined domains. The material displays a rough and heterogeneous texture, with filamentous or fibrous features arranged into an open and disordered network. No well-defined particle shape or uniform size distribution is observed. A similar morphology is found for PG200A10 (Figure 1c), where the fibrous texture is preserved but appears more extended and densely interconnected, indicating that prolonged treatment in air promotes further aggregation without producing rounded compact domains.

A different organization of the carbonaceous network is observed for the samples treated under static vacuum. PG200V5 (Figure 1b) is characterized by rounded, quasi-spherical aggregates with smoother external contours and lateral dimensions ranging from the sub-micrometer scale to several micrometers. These aggregates appear partially coalesced, forming clusters rather than isolated particles. Their internal contrast and surface texture suggest that the spherical domains are composed of intertwined filamentous or fibrous substructures, rather than being compact and featureless particles. After 10 h under vacuum, PG200V10 (Figure 1d) still shows rounded micrometer-scale aggregates with a textured internal appearance, suggesting that prolonged treatment preserves the filament-based organization while promoting further coalescence and densification. The overall SEM analysis indicates that both air and vacuum treatments generate carbonaceous materials with fibrous or filamentous substructures, but the reaction atmosphere strongly affects their mesoscale organization. Under air, these substructures form irregular and open aggregated networks, whereas under static vacuum they assemble into more compact quasi-spherical aggregates.

To evaluate whether temperature also plays a relevant role in the formation process, additional control experiments were carried out by heating phloroglucinol at 250 and 300 °C for 5 h under air or static vacuum. The SEM images reported in Figure S1 show that increasing the treatment temperature suppresses the filamentous aggregate morphology observed at 200 °C and promotes the formation of more continuous carbonaceous films. These samples display compact, wrinkled, and locally cracked surfaces, with sheet-like or lamellar features suggestive of more extended layered carbonaceous domains. No clear filamentous substructures are observed under these conditions.

The elemental composition of the samples treated at 200 °C was evaluated by energy-dispersive X-ray spectroscopy (EDX). The results, reported in Table S1, show a comparable carbon atomic percentage for PG200V5 compared with PG200A5 within the EDX experimental uncertainty. This is confirmed after 10 h, for which the air- and vacuum-treated samples display similar C/O ratios.

### 2.2. Raman/SERS Analysis and Structural Evolution

**Figure 2:**
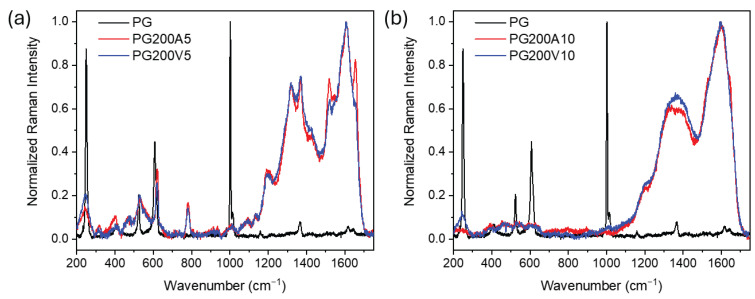
Normalized Raman spectra of pristine phloroglucinol (PG) and phloroglucinol-derived materials thermally treated at 200 °C under air or static vacuum: (**a**) comparison of PG, PG200A5, and PG200V5 after 5 h treatment; (**b**) comparison of PG, PG200A10, and PG200V10 after 10 h treatment.

The structural evolution of the samples was further investigated by Raman and SERS spectroscopy. Because of the intrinsically weak Raman signal of the thermally treated materials, SERS substrates were employed to improve the signal-to-noise ratio. The SERS measurements were used primarily to monitor the overall evolution of the vibrational features associated with the transformation of phloroglucinol into disordered carbonaceous structures. Since SERS enhancement depends on the local interaction between the analyte and the plasmonic substrate, the spectra were interpreted mainly in terms of band positions, broadening, and the appearance or disappearance of characteristic features rather than relative band intensities. Figure 2a shows the Raman spectra in the 200–1750 cm^−1^ range of pristine PG and of the samples obtained after thermal treatment at 200 °C for 5 h in air (PG200A5) or under static vacuum (PG200V5).

Pristine PG is dominated by sharp molecular vibrational modes, as expected for a crystalline molecular compound. In the 1050–1750 cm^−1^ region, only relatively weak and narrow features are observed, attributable to molecular aromatic vibrations of the precursor. In contrast, both PG200A5 and PG200V5 exhibit broad bands in the carbon-related spectral region. The feature centered around 1600 cm^−1^ can be assigned to the G band, associated with the in-plane stretching vibration of sp^2^ carbon atoms in aromatic or heteroaromatic domains.

In this context, the terms D-band and G-band are used to describe broad vibrational features characteristic of increasing aromatic condensation and disordered sp^2^-rich carbonaceous structures. They should not be interpreted as evidence for the formation of well-developed graphitic domains, which are not expected under the mild thermal treatment employed here (200 °C).

The 1200–1450 cm^−1^ region contains several overlapping contributions, including features around ∼1220, 1320, 1370, and 1430 cm^−1^. These bands can be associated with disorder-activated vibrational modes, including D-band-like contributions from small aromatic domains and vibrations involving oxygen-containing groups [40]. The complexity of this region suggests the formation of a heterogeneous carbonaceous network composed of aromatic, furanic, ether-like, and residual molecular motifs, in agreement with low-temperature carbonization. In the 5 h samples, some narrow features remain superimposed on the broader carbon-related bands, suggesting the presence of residual PG-like molecular units or molecular intermediates generated during thermal treatment.

The low-wavenumber region, between 200 and 1000 cm^−1^, further supports this structural evolution. Pristine PG shows intense and well-defined bands, including features at approximately 245, 620, and 1010 cm^−1^. After thermal treatment, these sharp molecular features are strongly attenuated or broadened, indicating a loss of long-range molecular order and the partial breakdown of the crystalline precursor structure. The disappearance or strong reduction of the intense band around ∼1010 cm^−1^ is particularly indicative of the transformation of PG into a structurally different carbonaceous material.

The treated samples also exhibit broad and weak features in the 315–410 cm^−1^ and 475–530 cm^−1^ regions, together with a residual contribution around ∼620 cm^−1^. These signals can be tentatively assigned to collective vibrational modes of disordered carbon–oxygen frameworks, including skeletal deformations of small aromatic or furanic units, C–O–C bending modes, and ring distortions typical of amorphous or polymerized carbonaceous structures. A weak feature around ∼780 cm^−1^, more evident in the air-treated sample, may be related to out-of-plane deformations of substituted aromatic rings or oxygen-containing heterocycles, suggesting a slightly larger contribution of oxygenated structural motifs when thermal treatment is performed in air [41].

When the treatment time is extended to 10 h (Figure 2b), the Raman spectra of PG200A10 and PG200V10 become very similar. Both samples show broad D- and G-like bands typical of disordered carbonaceous materials, while the narrow molecular features observed after 5 h are no longer evident. The low-wavenumber region also becomes less structured, with only weak and poorly resolved contributions. Similar Raman features are observed for samples treated at higher temperatures, 250 and 300 °C, as reported in Figure S2.

### 2.3. Steady-State and Time-Resolved Optical Properties

**Figure 3:**
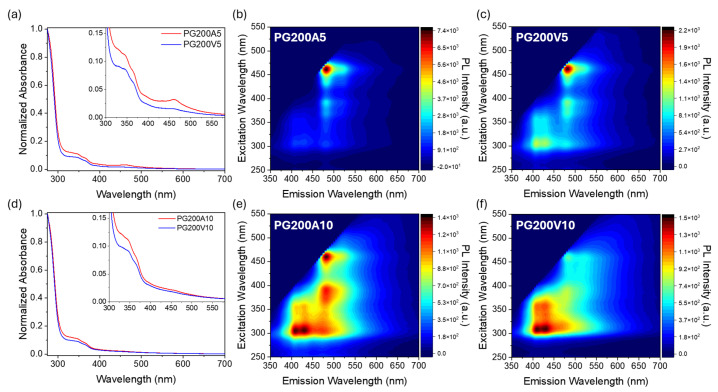
UV–Vis absorption spectra and photoluminescence excitation–emission maps of phloroglucinol-derived materials thermally treated at 200 °C under air or static vacuum. (**a**) Normalized UV–Vis absorption spectra of PG200A5 and PG200V5, with the inset highlighting the 300–580 nm region; (**b**,**c**) excitation–emission maps of PG200A5 and PG200V5, respectively; (**d**) normalized UV–Vis absorption spectra of PG200A10 and PG200V10, with the inset highlighting the 300–580 nm region; (**e**,**f**) excitation–emission maps of PG200A10 and PG200V10, respectively.

The optical absorption and photoluminescence properties of the PG-derived carbonaceous materials were investigated to evaluate how the reaction atmosphere and treatment time affect their electronic structure and emissive response. The normalized UV–Vis absorption spectra of the samples treated at 200 °C for 5 and 10 h under air or static vacuum are shown in Figure 3a,d. All samples display an intense absorption band in the UV region, centered at approximately 265 nm, together with a second contribution around 340 nm, followed by a gradual decrease toward the visible range. The high-energy absorption can be mainly associated with π–$\pi^{*}$ transitions of aromatic or sp^2^-rich domains derived from the phloroglucinol backbone, whereas the near-UV contribution is consistent with n–$\pi^{*}$ transitions involving oxygen-containing or defect-related states generated during thermal treatment. The insets in Figure 3a,d highlight the low-energy absorption region between 300 and 580 nm. A weak but distinguishable band around 460 nm is observed, especially in the samples treated for 5 h, and is more evident for PG200A5. After 10 h of treatment, this contribution decreases markedly and becomes barely detectable in the vacuum-treated sample.

**Figure 4:**
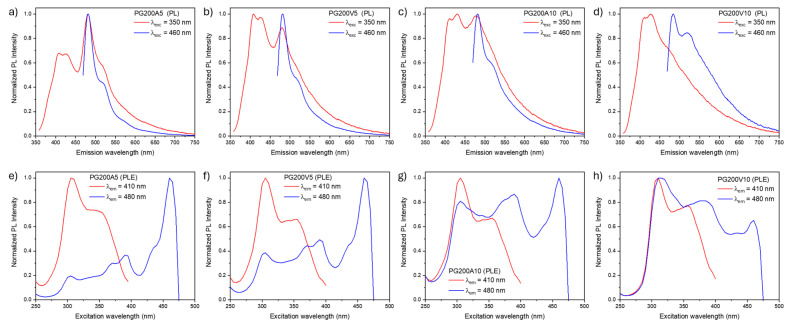
Normalized photoluminescence emission (PL) and excitation (PLE) spectra of phloroglucinol-derived materials thermally treated at 200 °C under air or static vacuum. (**a**–**d**) PL emission spectra of PG200A5, PG200V5, PG200A10, and PG200V10 recorded at excitation wavelengths of 350 and 460 nm. (**e**–**h**) PLE spectra of PG200A5, PG200V5, PG200A10, and PG200V10 monitored at emission wavelengths of 410 and 480 nm.

The photoluminescence excitation–emission maps (EEMs), reported in Figure 3b,c,e,f, were collected to compare the emissive features of the samples. Additional excitation and emission spectra extracted at selected wavelengths are shown in Figure 4. Overall, the EEMs reveal the presence of three main emissive contributions in the blue visible range. Two high-energy centers emit at approximately 410 and 425 nm and share excitation channels in the UV region, mainly around 310 and 350 nm. A third center gives rise to cyan emission centered at approximately 480 nm, with a main excitation channel around 460 nm and secondary excitation contributions in the UV region, around 310 and 380 nm.

The relative intensity of these emissive centers depends on the synthesis conditions. In PG200A5, the cyan emission is the dominant contribution, with an intensity significantly higher than that of the violet/deep-blue centers. Its relative contribution decreases under static vacuum and upon increasing the treatment time. In PG200V10, the violet and deep-blue emissions become the dominant contributions, whereas the cyan band is strongly reduced. The extracted PL and PLE spectra (Figure 4) support this assignment, showing that the cyan center can be spectrally distinguished from the higher-energy centers, which share similar excitation profiles.

Time-resolved photoluminescence measurements were carried out by exciting the samples at 360 and 460 nm, corresponding to the excitation regions of the violet/deep-blue and cyan emissive centers, respectively. The resulting decay curves, reported in Figure S3, are non-single-exponential, as commonly observed in heterogeneous fluorescent carbonaceous systems. The decay curves were satisfactorily described by a biexponential model. A fast decay component on the order of the instrumental time resolution (approximately 0.5–1 ns) and a slower component of approximately 3–4 ns were obtained for all samples. Since the fast component is comparable to the experimental temporal resolution, its absolute value should be regarded only as an approximate estimate. Nevertheless, no systematic differences in the overall decay behavior or in the slower nanosecond component were observed among the investigated samples, indicating that the synthesis conditions mainly modify the relative population of the emissive centers rather than producing major changes in their recombination dynamics.

### 2.4. Transient Absorption Spectroscopy

Transient absorption (TA) spectroscopy was used to investigate the excited-state dynamics associated with the emissive centers identified by steady-state optical measurements. Two pump wavelengths were selected: 360 nm, corresponding to the excitation region of the violet/deep-blue emissive centers, and 430 nm, within the low-energy absorption band associated with the cyan-emitting center. PG200A5 was selected as a representative sample because it displays the strongest cyan emission and the most clearly resolved transient spectral features.

**Figure 5:**
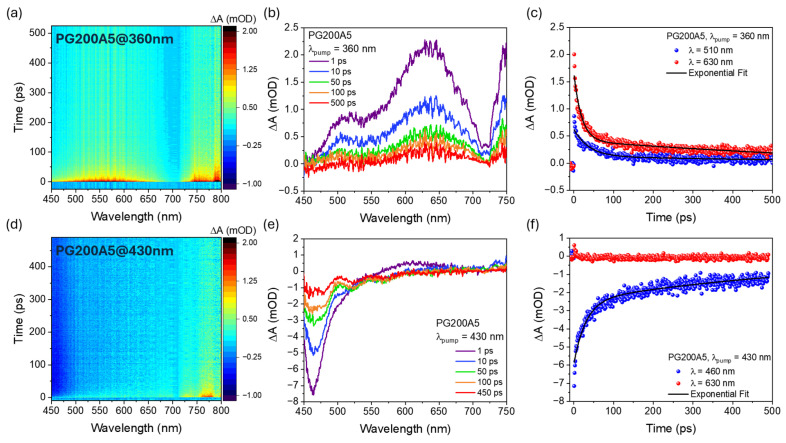
Transient absorption analysis of PG200A5. (**a**) Two-dimensional transient absorption map recorded after excitation at $\lambda_{\mathrm{pump}}=360$ nm. (**b**) Transient absorption spectra recorded at selected delay times after 360 nm excitation. (**c**) Kinetic traces extracted at representative probe wavelengths, $\lambda_{\mathrm{probe}}=510$ and 630 nm; black lines represent exponential fits of the experimental data. (**d**) Two-dimensional transient absorption map recorded after excitation at $\lambda_{\mathrm{pump}}=430$ nm. (**e**) Transient absorption spectra recorded at selected delay times after 430 nm excitation. (**f**) Kinetic traces extracted at $\lambda_{\mathrm{probe}}=460$ and 630 nm; black lines represent exponential fits of the experimental data.

The two-dimensional TA map of PG200A5 after 360 nm excitation is shown in Figure 5a. A broad positive excited-state absorption (ESA) signal extends over most of the investigated visible range. The spectra extracted at selected delay times (Figure 5b) show partially overlapping contributions, including a shoulder in the 500–550 nm region and a broader band centered around 630–650 nm. Both contributions decrease with increasing delay time, although a residual positive signal remains within the investigated 500 ps temporal window.

The corresponding kinetic traces extracted at 510 and 630 nm are reported in Figure 5c. Their non-single-exponential behavior indicates the presence of multiple relaxation processes. To resolve the fastest and longest components, the dynamics were fitted over different temporal windows. The analysis identifies an ultrafast component of a few picoseconds, an intermediate component on the tens-to-hundreds of picoseconds timescale, and a longer-lived contribution extending into the sub-nanosecond or nanosecond regime. The fitting parameters are reported in Table S2. These multicomponent dynamics are consistent with rapid relaxation from initially populated excited states, followed by trapping, interconversion, or decay of longer-lived excited-state populations.

A markedly different transient response is observed after excitation at 430 nm. The two-dimensional map in Figure 5d and the spectra in Figure 5e show an intense negative signal in the 450–550 nm region, accompanied by a much weaker positive contribution at longer wavelengths. The negative feature spectrally overlaps with both the absorption band around 460 nm and the cyan photoluminescence centered near 480 nm and can therefore be attributed to ground-state bleaching and/or stimulated emission associated with the cyan-emitting center.

The kinetic trace recorded at 460 nm (Figure 5f) exhibits multicomponent recovery, including a fast component of 1.1±0.1 ps, an intermediate component of approximately 80 ps, and a slower component of approximately 650 ps. By contrast, the weak positive signal monitored at 630 nm decays predominantly on a few-picosecond timescale. The corresponding fitting parameters are reported in Table S3. These results indicate that excitation at 430 nm directly populates the electronic states associated with the cyan-emitting center, which subsequently undergo ultrafast relaxation and sub-nanosecond recovery.

**Figure 6:**
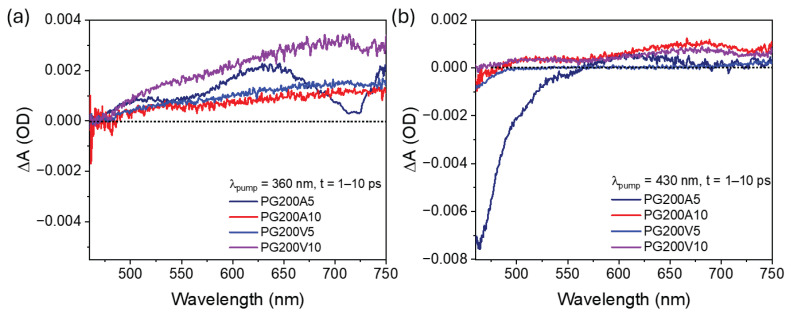
Comparison of the early-time transient absorption spectra of phloroglucinol-derived carbonaceous materials thermally treated at 200 °C under air or static vacuum for 5 and 10 h. Spectra were averaged over the 1–10 ps time window after excitation at (**a**) $\lambda_{\mathrm{pump}}=360$ nm and (**b**) $\lambda_{\mathrm{pump}}=430$ nm.

To compare the effects of treatment atmosphere and duration, the early-time TA spectra of all samples, averaged over the 1–10 ps interval, are shown in Figure 6. After 360 nm excitation (Figure 6a), all samples display positive ESA signals extending across the visible range, although their intensity and spectral shape depend on the synthesis conditions. PG200A5 and PG200V5 show relatively structured profiles, with contributions in the 500–550 and 600–700 nm regions. PG200A10 exhibits a weaker and less structured response, whereas PG200V10 displays the largest positive ESA amplitude, particularly above 600 nm.

The comparison after 430 nm excitation is shown in Figure 6b. PG200A5 exhibits a pronounced negative signal in the 450–550 nm region, consistent with the strong cyan absorption and emission observed in the steady-state measurements. A weaker negative contribution is also observed for PG200V5. In contrast, PG200A10 and PG200V10 show predominantly positive TA signals, with no clearly resolved negative feature in this spectral region. The disappearance or strong reduction of the negative signal after prolonged thermal treatment agrees with the reduced contribution of the cyan-emitting center observed in the excitation–emission maps.

The kinetic analyses of PG200A10, PG200V5, and PG200V10 are reported in Figure S4, while the corresponding fitting parameters are summarized in Tables S2 and S3. The probe wavelengths and integration ranges were selected according to the spectral regions displaying the most clearly resolved transient features and the highest signal-to-noise ratio. Despite differences in spectral amplitude and relative contributions, all samples exhibit multicomponent dynamics spanning ultrafast, intermediate, and longer-lived timescales. These results suggest that the synthesis atmosphere and treatment duration primarily modify the relative population and spectral weight of the excited-state species, whereas the overall relaxation pattern remains qualitatively similar.

### 2.5. Computational Modeling

The computational calculations were used to examine whether representative phloroglucinol-derived molecular motifs could account for the main optical features observed experimentally. Considering the relatively low treatment temperature, two simplified families of structures were initially investigated: oligomers formed by connected PG-derived units and conjugated structures containing an increasing number of furan-like bridges. Additional mixed configurations combining PG-derived and furan-containing units were also considered. These models are not intended to reproduce the full structural complexity of the carbonaceous materials, but rather to provide a molecular-level framework for interpreting the observed spectral features.

**Figure 7:**
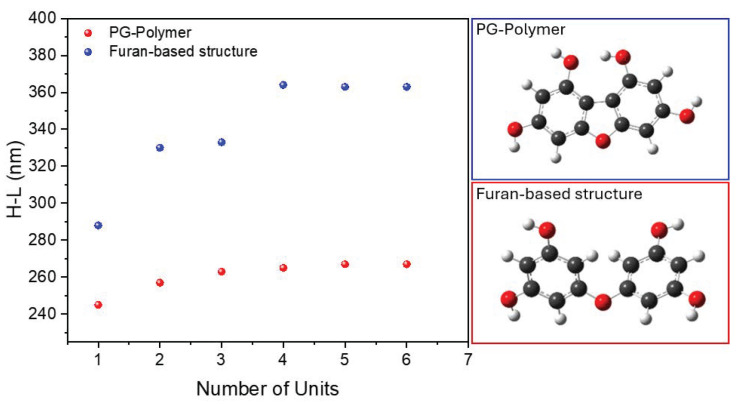
Wavelength equivalents of the calculated HOMO–LUMO energy gaps for the proposed PG-oligomer and furan-containing models as a function of the number of repeating units. Representative optimized structures of the two model families are shown on the right. The complete sets of optimized structures and corresponding calculated values are reported in Figures S5 and S6.

Figure 7 reports the wavelength equivalents of the calculated HOMO–LUMO energy gaps for the PG-oligomer and furan-containing series as a function of the number of repeating units. In both families, increasing the size of the conjugated structure produces a progressive red shift followed by an apparent saturation. The PG-oligomer series evolves from approximately 245 nm for the monomer to about 267 nm for the larger structures, whereas the furan-containing series shifts from approximately 288 nm to about 363–364 nm. The complete sets of optimized structures and corresponding calculated values are reported in Figure S5.

The larger red shift obtained for the furan-containing models indicates that furan-like connectivity lowers the electronic gap more effectively than the simple extension of PG-derived oligomers. These structures therefore represent plausible candidates for the molecular motifs contributing to the near-UV excitation channels of the violet and deep-blue emissive centers. However, the calculations do not provide a unique structural assignment, because different configurations may exhibit comparable electronic gaps.

Additional compact and mixed structures were therefore examined, as reported in Figure S6. The mixed PG/furan models display wavelength equivalents around 339–340 nm, indicating that changes in connectivity and branching can tune the electronic gap within the near-UV region. This result is consistent with the presence of a distribution of structurally related conjugated motifs contributing to the overlapping violet and deep-blue emission bands.

The compact triangular models show a stronger dependence on molecular size. The smaller T3 structure gives a calculated wavelength equivalent of approximately 340 nm, whereas the larger T6 model shifts to approximately 437 nm. This value approaches the low-energy absorption channel experimentally associated with the cyan-emitting center. The calculations therefore support the previously proposed assignment of the cyan emission to compact triangular PG-derived structures.

Finally, solvent effects were evaluated for two representative structures, namely a PG dimer and a model containing one furan-like bridge, using ethanol as the dielectric medium to reproduce the experimental conditions. Inclusion of the solvent environment produced no appreciable shift for the PG dimer and only a small blue shift of approximately 5 nm for the furan-containing model compared with the corresponding vacuum calculations. Within the limitations of the continuum-solvent approach, these results suggest that the calculated spectral trends are governed mainly by molecular connectivity and conjugation, while bulk solvent polarization plays a comparatively minor role.

## 3. Discussion

### 3.1. Atmosphere- and Temperature-Dependent Morphological Evolution

The SEM results indicate that the treatment atmosphere primarily affects the mesoscale organization of the phloroglucinol-derived material at 200 °C. The relatively mild thermal treatment employed in the present work (200 °C) is expected to promote only partial carbonization, preserving a significant fraction of oxygen-containing functional groups and molecularly defined aromatic motifs. As a consequence, the resulting materials retain localized electronic states that support excitation-dependent photoluminescence and multiple emissive centers. In contrast, considerably higher carbonization temperatures typically promote extensive aromatization and condensation into larger sp^2^ domains, progressively suppressing localized molecular states while favoring the formation of continuous electronic pathways responsible for enhanced electrical conductivity and mechanical robustness. Therefore, the present synthetic approach is not intended to produce highly graphitized carbon frameworks but rather to generate partially carbonized materials whose heterogeneous electronic structure is particularly attractive for optical and photophysical applications. Both air- and vacuum-treated samples display filamentous or fibrous substructures, but these features assemble differently depending on oxygen availability. Under air, they form irregular, open, and highly interconnected aggregates, whereas under static vacuum they organize into more compact quasi-spherical domains. The distinction therefore does not appear to arise from the formation of entirely different primary structural elements, but rather from their different degree of aggregation, coalescence, and spatial organization.

This behavior suggests that the surrounding atmosphere modifies the balance among cross-linking, condensation, oxidative reactions, and structural relaxation during the early stages of PG thermal evolution. Under air, oxidative pathways may increase the number of competing reaction channels and favor spatially heterogeneous cross-linking, thereby limiting the formation of compact domains. Under static vacuum, the suppression of oxidative side reactions may instead allow filamentous fragments to reorganize and coalesce into more clearly defined rounded aggregates [14,42].

Increasing the treatment temperature to 250–300 °C produces a more pronounced morphological transformation. The filamentous aggregates observed at 200 °C are replaced by compact, wrinkled, and locally cracked film-like structures with sheet-like or lamellar features. The crack patterns are consistent with shrinkage and mechanical stress release during thermal consolidation. At these higher temperatures, the similarities between air- and vacuum-treated samples become more evident, suggesting that temperature-driven coalescence increasingly dominates over the atmosphere-dependent organization observed at 200 °C.

The EDX analysis provides complementary, although semi-quantitative, information on the elemental composition of the samples. Within the experimental uncertainty inherent to EDX measurements on heterogeneous carbonaceous powders, all samples exhibit comparable carbon and oxygen contents, indicating that the thermal treatments carried out under air and static vacuum produce materials with essentially similar overall elemental compositions. These results suggest that the different reaction atmospheres primarily influence the structural organization and optical properties of the materials rather than inducing substantial changes in their bulk elemental composition.

### 3.2. Structural Evolution Monitored by Raman Spectroscopy

Raman spectroscopy shows that treatment at 200 °C is sufficient to induce a marked transformation of crystalline phloroglucinol. After 5 h, both air- and vacuum-treated samples exhibit broad carbon-related features superimposed with narrower molecular contributions. The broad bands in the 1200–1650 cm^−1^ region are consistent with the formation of small, disordered sp^2^-rich aromatic or heteroaromatic domains, whereas the residual narrow modes indicate that PG-like units or molecular intermediates are still present.

The spectra therefore support a gradual rather than abrupt conversion of PG into a carbonaceous network. At this relatively low temperature, the resulting material is expected to remain chemically and structurally heterogeneous, containing small conjugated domains connected through oxygen-containing and partially polymerized motifs.

After 10 h, the narrow molecular features disappear and the spectra of PG200A10 and PG200V10 become very similar. This convergence indicates that prolonged treatment promotes further structural reorganization and reduces the differences initially introduced by the atmosphere. Importantly, the broad D- and G-like features are more consistent with small disordered sp^2^-rich domains than with extended graphitic order. The Raman data therefore support the formation of an increasingly condensed carbonaceous framework, but do not provide evidence for highly graphitized or nanocrystalline graphite-like structures [10,25,40,43].

Similar broad Raman features are also observed after treatment at 250 and 300 °C. This suggests that prolonged treatment at 200 °C can produce a local bonding environment comparable, at the level probed by Raman spectroscopy, to that obtained at higher temperatures, even though the corresponding mesoscale morphologies remain different. These results indicate that prolonged thermal treatment promotes further evolution from the molecular precursor toward a disordered carbonaceous network and reduces the differences initially induced by the reaction atmosphere.

### 3.3. Multiple Emissive Centers and Their Dependence on Synthesis Conditions

The optical data reveal that the PG-derived materials contain more than one optically active species or structural motif. The excitation–emission maps distinguish two closely spaced high-energy contributions, emitting at approximately 410 and 425 nm, and a separate cyan-emitting center around 480 nm [23,25,27]. The violet and deep-blue centers share excitation channels in the UV region, whereas the cyan center is most efficiently excited near 460 nm and also displays weaker excitation channels at higher energy.

The relative contributions of these centers vary systematically with synthesis conditions. PG200A5 displays the strongest cyan emission and the most pronounced absorption feature around 460 nm. The cyan contribution decreases both under static vacuum and with increasing treatment time, becoming strongly reduced in PG200V10. By contrast, the violet and deep-blue components become relatively more important after prolonged treatment, particularly under vacuum.

These changes indicate that atmosphere and treatment duration modulate the relative abundance or optical strength of the emissive motifs rather than producing completely different photophysical species. This interpretation is supported by the time-resolved PL measurements. Although the relative emission intensities change substantially, all samples display comparable biexponential decay components of approximately 0.5–1.0 and 3–4 ns. The comparable overall decay profiles, together with the similar values obtained for the slower nanosecond component, suggest that the synthesis conditions primarily affect the relative abundance of the emissive centers rather than introducing markedly different recombination pathways.

The stronger cyan contribution in PG200A5 is therefore more plausibly attributed to a larger relative population of the corresponding emissive motif than to the appearance of a distinct recombination mechanism. Likewise, the enhanced violet/deep-blue contribution in the longer-treated and vacuum-treated samples reflects a redistribution among the optically active structures formed during thermal evolution.

### 3.4. Excited-State Dynamics and the Multi-Center Photophysical Model

Transient absorption spectroscopy provides further evidence for the coexistence of multiple excited-state populations. Under 360 nm excitation, PG200A5 exhibits a broad positive ESA extending across most of the visible range, with partially overlapping contributions around 500–550 and 630–650 nm. The non-single-exponential kinetics reveal relaxation over multiple timescales, including a few-picosecond component, an intermediate tens-to-hundreds-of-picoseconds component, and a longer-lived sub-nanosecond or nanosecond contribution [25,27].

The ultrafast component can be associated with relaxation from initially populated high-energy states, including vibrational relaxation, local structural relaxation, solvation, or rapid transfer toward lower-energy excited states. The intermediate component is consistent with the redistribution of excited-state population among energetically related emissive states. At the present stage, the transient absorption data do not allow us to distinguish whether this reflects energy transfer between spatially distinct motifs, relaxation within different regions of a larger conjugated structure, or other excited-state relaxation processes. Therefore, the proposed model should be regarded as a phenomenological description of the observed dynamics rather than as evidence for a specific microscopic mechanism. These assignments are not unique, but they provide a physically plausible interpretation of the multicomponent kinetics observed in a structurally heterogeneous carbonaceous system. The longest TA component approaches the timescale of the PL decays and is therefore consistent with the population of states involved in radiative recombination.

Excitation at 430 nm produces a qualitatively different response. In PG200A5, the strong negative feature between 450 and 550 nm spectrally overlaps both the low-energy absorption band and the cyan emission. It can therefore be assigned to ground-state bleaching and/or stimulated emission associated with the cyan-emitting center. Its multicomponent recovery, including approximately 1, 80, and 650 ps components, indicates that the selectively populated low-energy states undergo both ultrafast relaxation and longer-lived recovery.

The cyan-related negative feature is strongly reduced in PG200V5 and is not clearly resolved in the 10 h samples. This trend mirrors the steady-state PL results and indicates that prolonged treatment decreases the relative population or transition strength of the cyan-emitting motif. In contrast, the positive ESA contribution remains present in all samples, although its intensity and spectral distribution change. PG200V10 displays the strongest positive ESA above 600 nm under 360 nm excitation, consistent with the increased relative importance of the violet/deep-blue centers observed in the EEMs.

Taken together, the TA data indicate that synthesis conditions mainly redistribute the relative spectral weight of the excited-state populations. The overall relaxation pattern remains qualitatively similar, with ultrafast relaxation followed by intermediate trapping or interconversion and longer-lived decay. Oxygen availability therefore appears to affect the relative abundance of the emissive motifs more strongly than the fundamental timescales governing their relaxation.

### 3.5. Computational Support for the Structural Assignments

The computational models provide a molecular-level framework for interpreting the experimentally observed excitation channels. The calculations do not uniquely identify the structures present in the samples, but they show how changes in connectivity, conjugation length, and molecular topology can shift the electronic gap over the experimentally relevant spectral range.

It should be emphasized that the calculated Kohn–Sham HOMO-LUMO gaps are not intended to reproduce the experimental optical transition energies quantitatively. Rather, they are used here as qualitative descriptors of the relative electronic conjugation of the proposed structural motifs. Since excited-state effects, including electron-hole interactions and orbital relaxation, are not explicitly accounted for within this approach, only qualitative comparisons with the experimentally observed excitation channels are appropriate. The calculated gaps correctly reproduce the relative ordering of the candidate structures and therefore provide useful support for assigning the different emissive centers to chemically distinct molecular motifs.

For the PG-oligomer series, the wavelength equivalent of the HOMO–LUMO gap saturates near 267 nm, whereas the furan-containing series reaches approximately 363–364 nm. Furan-like connectivity therefore lowers the electronic gap more efficiently than simple extension of PG-derived oligomeric chains. This makes the furan-containing models plausible candidates for motifs contributing to the near-UV excitation channels of the violet and deep-blue emissive centers.

The mixed PG/furan structures exhibit calculated values around 339–340 nm. Their similar but non-identical electronic gaps indicate that variations in branching and connectivity can generate a distribution of closely spaced excited states. Such a distribution is consistent with the overlapping violet and deep-blue emission bands and with the broad ESA response observed under 360 nm excitation. Rather than requiring two completely unrelated chemical species, the optical data may therefore reflect a family of structurally related oxygen-containing conjugated motifs [34].

The triangular structures show a stronger size dependence. The smaller T3 model gives a wavelength equivalent near 340 nm, whereas the larger T6 structure reaches approximately 437 nm, close to the low-energy absorption channel associated with the cyan emission. This result supports the previously proposed assignment of the cyan-emitting center to compact triangular PG-derived structures, although it does not constitute a unique structural identification [27].

The solvent effect was evaluated using the IEFPCM continuum model with ethanol as the dielectric medium. The resulting variations in the calculated HOMO-LUMO gaps were limited (within approximately 5 nm in terms of the corresponding excitation wavelength). Nevertheless, it should be emphasized that this approach provides only a simplified description of the local environment. In particular, it does not account for specific solute-solvent interactions, hydrogen bonding, aggregation effects, or the heterogeneous surface states expected in the experimentally obtained carbonaceous materials. Consequently, the calculations should be regarded as providing qualitative insight into the intrinsic electronic properties of representative molecular motifs rather than a quantitative description of the complete condensed-phase system.

The minor solvent-induced shifts calculated in ethanol indicate that the general spectral trends are governed mainly by molecular connectivity and conjugation. Within the limitations of the continuum-solvent model, solvent polarization does not appear to be the primary origin of the differences among the experimentally observed emissive centers.

Taken together, the computational results should be interpreted as a qualitative framework for rationalizing the experimental observations. The calculated electronic gaps are intended to compare the relative electronic structure of representative molecular motifs rather than to quantitatively reproduce the optical properties of the heterogeneous carbonaceous material. The combined use of experimental spectroscopy and DFT therefore allows the identification of plausible emissive motifs while acknowledging that the actual material contains a broader structural and environmental complexity than can be represented by the present molecular models.

### 3.6. Proposed Structure–Photophysics Relationship

The combined results support a model in which low-temperature PG treatment generates a structurally heterogeneous ensemble of conjugated motifs rather than a uniform carbon phase. PG-derived oligomeric fragments, furan-containing domains, mixed PG/furan structures, and compact triangular motifs may coexist within the same carbonaceous material. Their relative populations depend on oxygen availability and treatment duration.

The violet and deep-blue emissions are tentatively associated with families of furan-containing and mixed conjugated domains displaying closely spaced electronic gaps. The cyan emission is instead associated with a more compact triangular motif absorbing in the 430–460 nm region. Thermal treatment changes the balance among these structures: prolonged heating decreases the cyan contribution and increases the relative weight of the higher-energy emissive centers, particularly under static vacuum.

More broadly, the ability to modulate the relative distribution of emissive centers through simple control of the reaction atmosphere provides a potential strategy for tailoring the optical response of low-temperature carbonaceous materials. Such control may prove valuable for applications requiring tunable fluorescence, including optical encoding, anti-counterfeiting technologies, chemical sensing, and imaging, while also contributing to a deeper understanding of the processing–structure–property relationships governing emissive carbonaceous systems.

**Figure 8:**
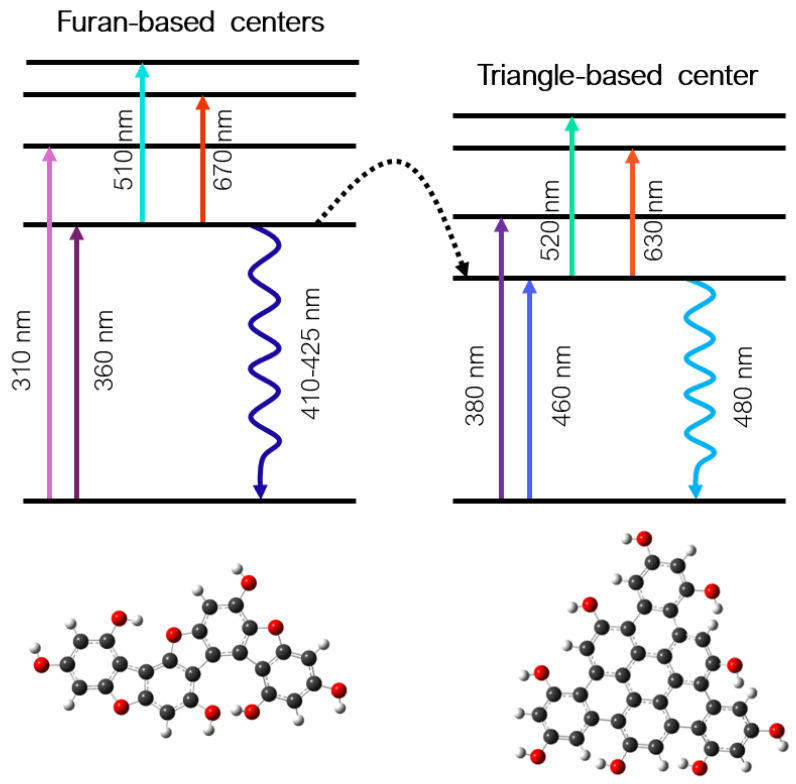
Schematic representation of the proposed photophysical model for phloroglucinol-derived carbonaceous materials. Furan-containing and structurally related conjugated motifs are associated with the violet and deep-blue emissive contributions, whereas compact triangular PG-derived motifs are proposed as candidates for the cyan-emitting center. The diagram summarizes the principal excitation and emission channels and a possible possible excited-state relaxation pathway connecting the two different emissive motifs. Representative optimized molecular structures are shown for each family.

Figure 8 summarizes this proposed photophysical picture. The scheme should be regarded as a simplified representation of the dominant excitation and emission pathways rather than as a complete energy-level diagram. The dashed connection between the two families of emissive centers is intended only as a schematic representation of a possible relationship between the corresponding excited-state populations inferred from the transient absorption measurements. It should not be interpreted as evidence for a specific microscopic transfer mechanism.

## 4. Materials and Methods

### 4.1. Sample Preparation

1,3,5-Trihydroxybenzene, commonly referred to as phloroglucinol (PG), supplied by Merck Ltd. (Merck Life Science srl, Milano Italy) with a purity of ≥99.0%, was used as the sole precursor for the preparation of carbonaceous materials. The pristine PG powder was used without further purification. Thermal treatments were carried out in a tubular furnace at 200 °C for 5 or 10 h, using a heating rate of 10 °C/min and allowing the furnace to cool naturally to room temperature. Powder samples (100 mg) were ground and placed in open or sealed quartz tubes, 10 cm long and 1 cm in diameter. The pyrolysis experiments were performed either under atmospheric open-air conditions or under a static vacuum regime, with a pressure lower than $3.0\times 10^{-5}$ Torr.

### 4.2. Experimental Set-Up

Scanning Electron Microscopy (SEM) images were acquired using an ESEM FEI Quanta 200 microscope (FEI Company, Hillsboro, OR, USA), with a maximum lateral resolution of 4 nm. The elemental composition of the samples was determined by X-ray microanalysis using an energy-dispersive X-ray spectroscopy detector (EDS).

Structural information was obtained by Surface-Enhanced Raman Spectroscopy (SERS) measurements performed in backscattering geometry using a Micro Raman confocal scattering system, SOL Confotec MR750, SOL Instruments Ltd., Minsk, Republic of Belarus, equipped with a Nikon Eclipse Ni microscope, Nikon Instruments Europe BV, Amsterdam, The Netherlands. The excitation wavelength was 532 nm, provided by an IO MatchBox series laser diode (Integrated Optics, Vilnius, Vilniaus Apskritis, Lithuania) with an excitation power of 3 mW. The signal was collected with a spectral resolution of 0.6 cm^−1^, using a sensor temperature of −24 °C, an Olympus 50× objective (Evident Corporation, Tokyo, Japan), and a grating with 1200 grooves/mm. Indium tin oxide (ITO) glass substrates coated with silver nanoparticles were used as SERS supports, namely S-Silver SERS substrates, Sersitive, Warsaw, Poland. The reference spectrum of pure PG powder was collected under standard Raman spectroscopy conditions using 785 nm excitation from an IO MatchBox series laser diode, with an excitation power of 3 mW, without SERS supports.

Steady-state optical absorption measurements were performed using a Jasco V-750 spectrophotometer (JASCO Corporation, Ishikawa-machi, Hachioji, Tokyo, Japan) in the 200–800 nm spectral range, with a spectral bandwidth of 0.2 nm. The samples were diluted in ethanol and placed in quartz cuvettes with a 1 cm optical path length. Baseline corrections were applied to all spectra.

The excitation and emission optical features were investigated by collecting three-dimensional excitation–emission maps (EEMs) using a Jasco FP-8550 spectrofluorometer (JASCO Corporation, Ishikawa-machi, Hachioji, Tokyo, Japan). The samples were excited in the 250–550 nm range, with an excitation spectral bandwidth of 5 nm, using a 450 W xenon lamp as the excitation source, Horiba Ltd., Kyoto, Japan. The photoluminescence emission was recorded in the 275–750 nm range, with an emission spectral bandwidth of 1 nm.

Time-resolved photoluminescence (TR-PL) measurements were carried out by exciting the samples with 200 fs laser pulses generated by an optical parametric amplifier, TOPAS-C, Light Conversion (Vilnius, Lithuania), pumped by a regenerative Ti:sapphire amplifier, Coherent Libra-HE (Coherent Inc., Santa Clara, CA, USA). The laser system operated at a repetition rate of 1 kHz. The photoluminescence emission was detected using a Hamamatsu C10910 streak camera (Hamamatsu Photonics, Hamamatsu City, Shizuoka Pref., Japan) coupled to an Acton SpectraPro SP-2300 grating spectrometer, Princeton Instruments (Teledyne Princeton Instruments, Trenton, NJ, USA). All measurements were performed on solutions contained in quartz cuvettes with a 1 cm optical path length, and the emission was collected in front-face geometry. When required, suitable optical filters were used to suppress scattered excitation light. The overall temporal resolution of the streak-camera measurements in the reported experiments was approximately 0.5 ns, estimated from the 10–90% rise time of the emission signal.

Transient absorption (TA) measurements were performed using a pump–probe set-up, HELIOS-80000-UV–VIS–NIR, Ultrafast Systems, Sarasota, FL, USA. The laser source was a regenerative Ti:sapphire amplifier, Libra-HE, Coherent (Coherent Inc., Santa Clara, CA, USA), delivering 200 fs pulses at 800 nm with a repetition rate of 1 kHz. The output pulse train was split to generate the pump and probe beams. The pump pulses were tunable in the 300–800 nm spectral range through an optical parametric amplifier, TOPAS-800-fs-UV-1 (Light Conversion, Vilnius, Lithuania), while the probe pulses consisted of a white-light supercontinuum generated by focusing the fundamental beam onto a sapphire plate. The temporally delayed pump and probe beams were spatially overlapped and focused onto a 1 mm path-length quartz cuvette containing an ethanolic dispersion of the phloroglucinol-derived carbonaceous material with an optical density below 0.5. All measurements were carried out at room temperature. No pump-intensity-dependent dynamics were observed within the investigated excitation fluence range, 0.1–0.6 mJ cm^−2^.

### 4.3. Simulations

The optimized structures and the corresponding self-consistent field (SCF) energies were calculated using a Density Functional Theory (DFT) approach with the Gaussian 16 suite of programs [44]. Calculations were performed at the B3LYP/6-311++G(d,p) level of theory [45,46]. Computational simulations were carried out either in vacuum or by including ethanol as solvent. Solvent effects were taken into account through a self-consistent reaction field approach, in which the dielectric environment was described using the polarizable continuum model (PCM) within the integral equation formalism variant (IEFPCM) [47].

UV–visible absorption spectra were simulated by calculating vertical electronic transitions, with the solvent environment treated consistently with the corresponding ground-state geometry. All optimized ground-state structures were confirmed to be true energy minima, as demonstrated by the absence of imaginary frequencies in the vibrational analyses. The calculated Raman vibrational modes were scaled using a quadratic scaling factor, as previously reported [48,49,50].

## 5. Conclusions

This work investigated the low-temperature thermal evolution of phloroglucinol under air and static vacuum by combining morphological, structural, steady-state and time-resolved optical measurements with transient absorption spectroscopy and computational modeling. At 200 °C, the treatment atmosphere primarily affects the mesoscale organization of the resulting carbonaceous materials. Air treatment produces irregular and open networks of filamentous substructures, whereas static vacuum favors their assembly into more compact quasi-spherical aggregates. At 250–300 °C, the filamentous morphology is replaced by compact, wrinkled, and locally cracked film-like structures, indicating that temperature-driven consolidation becomes increasingly dominant. EDX and Raman results further suggest that atmospheric effects are more pronounced during the early stages of thermal evolution, while prolonged treatment drives the air- and vacuum-treated samples toward a more similar disordered carbonaceous framework composed of small sp^2^-rich domains.

The optical response arises from multiple emissive centers whose relative contributions depend on atmosphere and treatment duration. Violet and deep-blue emissions are observed together with a distinct cyan contribution, which is most intense in PG200A5 and decreases under static vacuum and after prolonged treatment. Despite these changes in relative intensity, the photoluminescence lifetimes remain broadly comparable among the samples, indicating that the synthesis conditions mainly modify the relative population or transition strength of the emissive motifs rather than their fundamental recombination pathways. Transient absorption spectroscopy supports this multi-center picture, revealing ultrafast relaxation on the picosecond timescale, intermediate trapping or interconversion processes, and longer-lived sub-nanosecond or nanosecond decay. Excitation at 430 nm preferentially addresses the cyan-emitting center, as indicated by the ground-state-bleaching and/or stimulated-emission signal observed in the 450–550 nm region.

Computational calculations provide a plausible molecular-level framework for interpreting these optical features. Furan-containing and mixed PG/furan conjugated structures display electronic gaps compatible with the near-UV excitation channels of the violet and deep-blue centers, whereas larger compact triangular PG-derived motifs approach the low-energy absorption region associated with the cyan emission. Although these models do not uniquely identify the structures present in the samples, they support a picture in which low-temperature treatment generates a heterogeneous ensemble of structurally related conjugated motifs. The proposed molecular motifs should therefore be regarded as plausible structural models that rationalize the experimental observations rather than as definitive structural identifications of the emissive centers. Overall, oxygen availability and treatment duration modulate both the mesoscale morphology and the relative distribution of optically active structures in phloroglucinol-derived carbonaceous materials, providing a coherent structure–photophysics relationship for this low-temperature synthesis route.

## Data Availability

The original contributions presented in this study are included in the article/Supplementary Materials. Further inquiries can be directed to the corresponding author.

## Supplementary Materials

The following supporting information can be downloaded at: https://www.mdpi.com/article/10.3390/ijms27156632/s1.
