# Supplementary material for: Low-Temperature Formation and Photophysics of Phloroglucinol-Derived Carbonaceous Materials Under Air and Vacuum
Source: Int J Mol Sci. 2026 Jul 25;27(15):6632. doi: 10.3390/ijms27156632 (PMC13466324; doi:10.3390/ijms27156632)
Supplement: Supplementary file 1 [file ijms-27-06632-s001.zip › ijms-4456730-supplementary.pdf]

Supporting Information

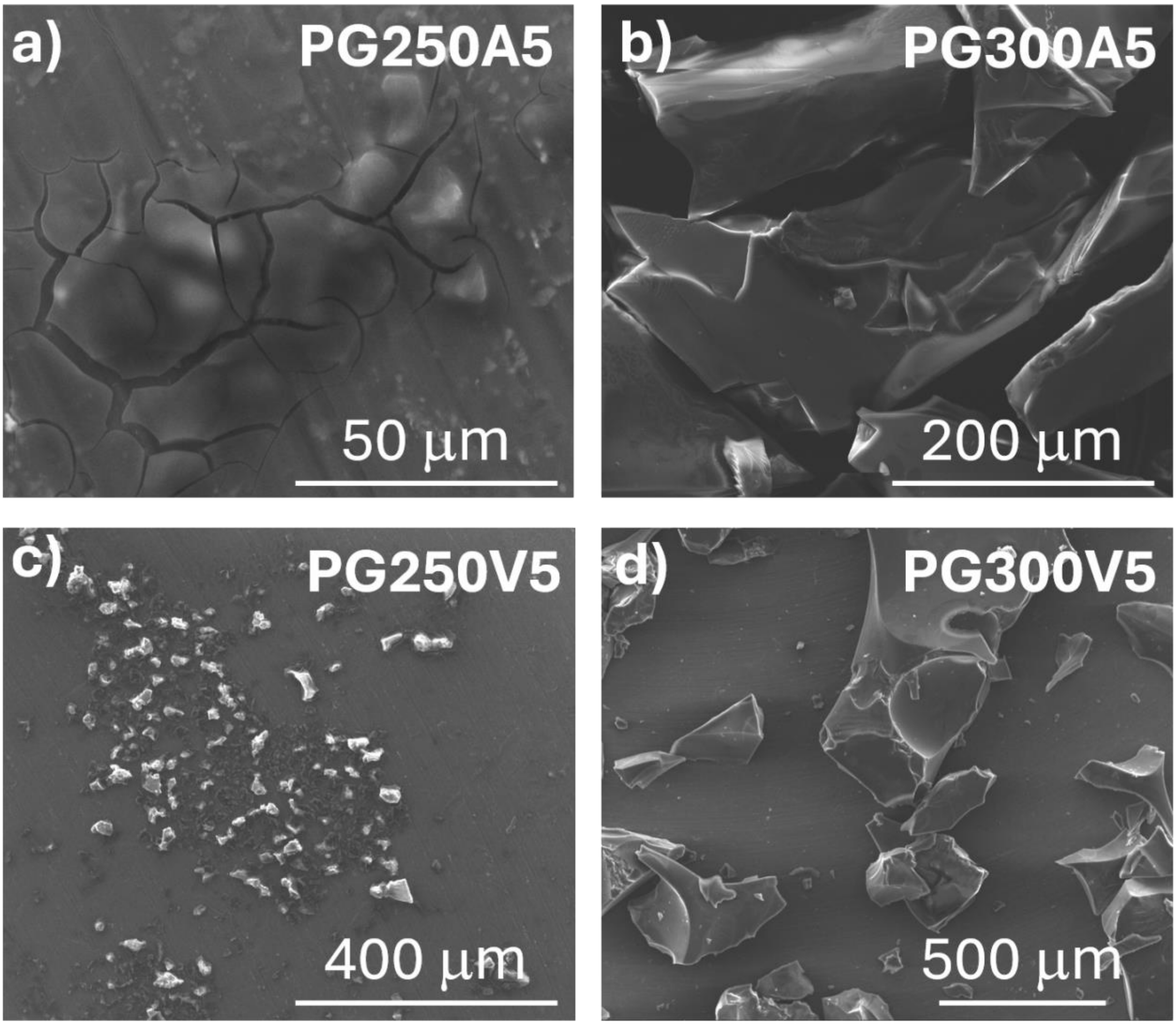

Figure S1. SEM micrographs of phloroglucinol-derived materials obtained after thermal treatment at higher temperatures under different atmospheres: (a) PG250A5, treated in air at 250 °C for 5 h; (b) PG300A5, treated in air at 300 °C for 5 h; (c) PG250V5, treated under static vacuum at 250 °C for 5 h; and (d) PG300V5, treated under static vacuum at 300 °C for 5 h.

Table S1. Atomic percentages of carbon and oxygen obtained by EDX analysis for phloroglucinol-derived materials thermally treated at 200 °C under air or static vacuum for 5 and 10 h.

	PG200A5	PG200V5	PG200A10	PG200V10
**C (atom %)**	70,6	72,0	68,9	68,2
**O (atom (%)**	29,4	28,0	31,1	31,8

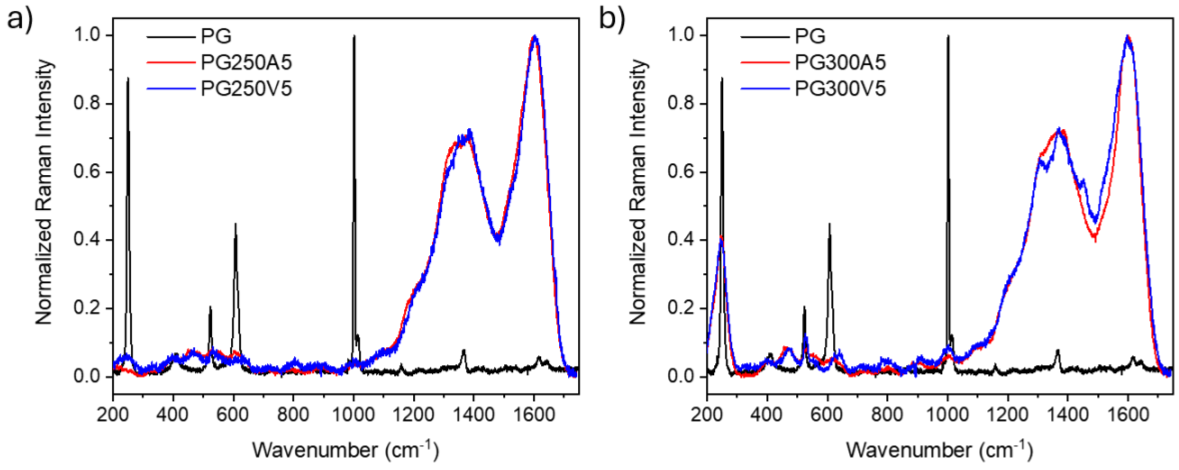

Figure S2. Normalized Raman spectra of pristine phloroglucinol (PG) and phloroglucinol-derived materials thermally treated for 5 h at 250 °C and 300 °Cunder air or static vacuum: **(a)** comparison of PG, PG250A5, and PG250V5; **(b)** comparison of PG, PG300A5, and PG300V5.

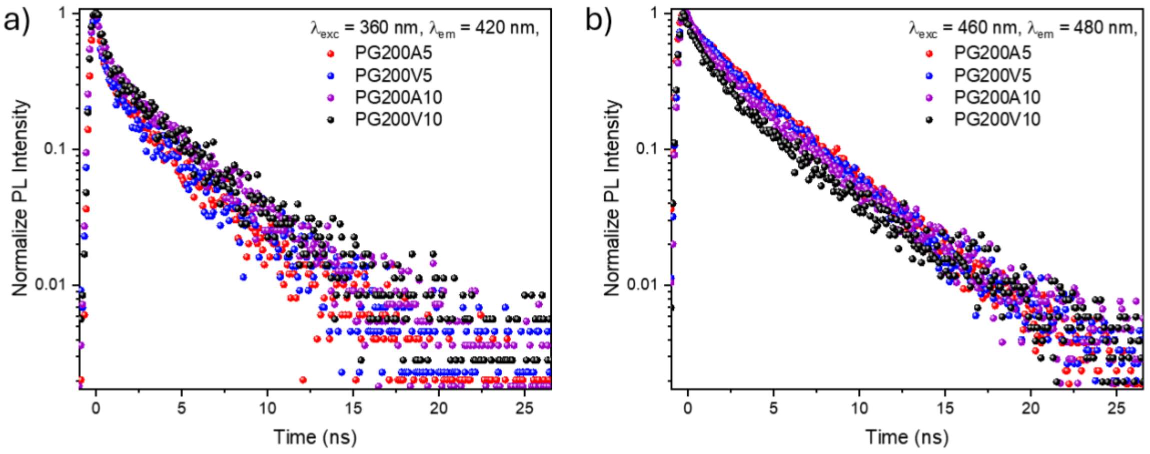

Figure S3. Time-resolved photoluminescence decay curves of phloroglucinol-derived materials thermally treated at 200 °C under air or static vacuum for 5 and 10 h. Decays were recorded at (a) λ_{exc} = 360 nm and λ_{em} = 420 nm, and (b) λ_{exc} = 460 nm and λ_{em} = 480 nm.

Table S2. Fitting parameters obtained from the transient absorption dynamics of phloroglucinol-derived materials thermally treated at 200 °C under air or static vacuum for 5 and 10 h. The data were extracted after excitation at 360 nm by monitoring two excited-state absorption regions, ESA1 (450–600 nm) and ESA2 (600–750 nm). Δt indicates the selected temporal window used for the fitting, while τ_1 and τ_2 represent the fast and slow decay components, respectively.

PUMP = 360 nm	Δt (ps)	ESA1 (450-600 nm)	ESA2 (600-750 nm)
**PG200A5**	500	τ_1 = 38±2; τ_2 = 1600±300;	τ_1 = 19,4±0,7; τ_2 = 1050±50
	50	τ_1 = 3,5±0,2; τ_2 = 138±6	τ_1 = 2,9±0,1; τ_2 = 128±3
**PG200A10**	1000		τ_1 = 46±3; τ_2 = 1480±160
	30		τ_1 = 2,5±0,3; τ_2 = 58±5
**PG200V5**	250		τ_1 = 18,3±0,6; τ_2 = 770±40
	30		τ_1 = 3,0±0,3; τ_2 = 54±3
**PG200V10**	1000	τ_1 = 37,2±1,5; τ_2 = 7800±600	τ_1 = 48,5±1,5; τ_2 = 1640±90
	100	τ_1 = 4,6±0,2; τ_2 = 84±3	τ_1 = 3,7±0,2; τ_2 = 64,2±1,6

Table S3. Fitting parameters obtained from the transient absorption dynamics of phloroglucinol-derived materials thermally treated at 200 °C under air or static vacuum for 5 and 10 h. The data were extracted after excitation at 430 nm by monitoring ground-state bleaching, GSB, at 450 nm and two excited-state absorption regions, ESA1 (450–600 nm) and ESA2 (600–750 nm). Δt indicates the selected temporal window used for the fitting, while τ_1 and τ_2 represent the fast and slow kinetic components, respectively.

PUMP = 430 nm	Δt (ps)	GSB (450 nm)	ESA1 (450-600 nm)	ESA2 (600-750 nm)
**PG200A5**	500	τ_1 = 30±1; τ_2 = 650±30		
	50	τ_1 = 1,1±0,1; τ_2 = 79±1		τ_1 = 5,7±0,5
**PG200A10**	3000			τ_1 = 33±15; τ_2 = 5600±500

	50			τ_1 = 4,2±0,3; τ_2 = 67±2
**PG200V5**	250	τ_1 = 27±2; τ_2 = 460±50		
	30	τ_1 = 0,7±0,3; τ_2 = 66±7		
**PG200V10**	1000		τ_1 = 26±10; τ_2 = 3700±600	τ_1 = 39±10; τ_2 = 10400±1700
	50		τ_1 = 3,1±0,5; τ_2 = 65,9±0,9	τ_1 = 2,5±0,5; τ_2 = 58,9±0,5

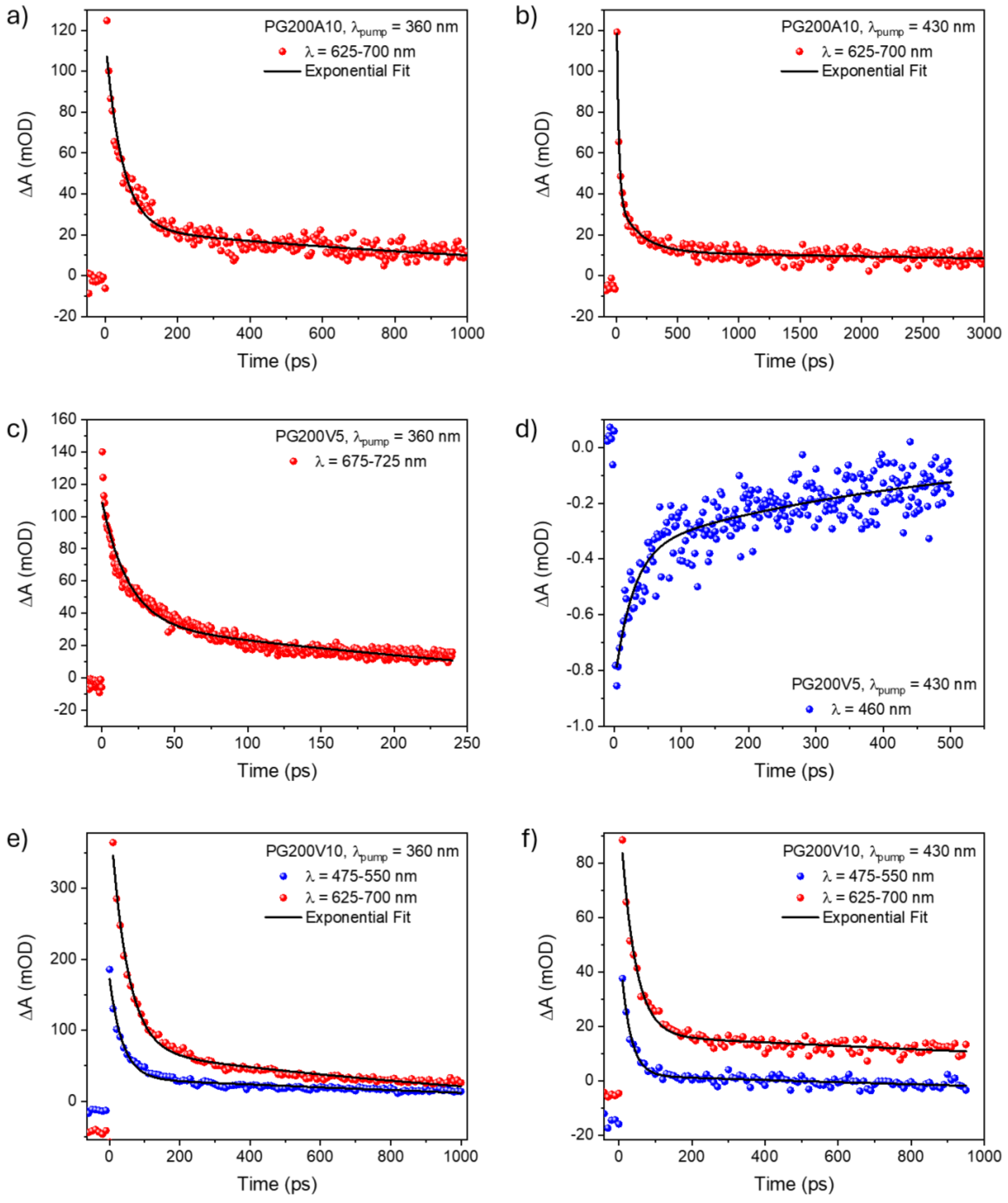

Figure S4. Transient absorption kinetic traces of phloroglucinol-derived carbonaceous materials thermally treated at 200 °C under air or static vacuum. (a,b) Dynamics of PG200A10 after excitation at λ_pump = 360 and 430 nm, respectively, obtained by integrating the transient absorption signal over the 625–700 nm spectral region. (c,d) Dynamics of PG200V5 after excitation at λ_pump = 360 and 430 nm, respectively; the traces were extracted by integrating the signal over the 675–725 nm spectral region in panel (c) and at λ_probe = 460 nm in panel (d). (e,f) Dynamics of PG200V10 after excitation at λ_pump = 360 and 430 nm, respectively, obtained by integrating the signal over the 475–550 and 625–700 nm spectral regions. Black lines represent exponential fits of the experimental data.

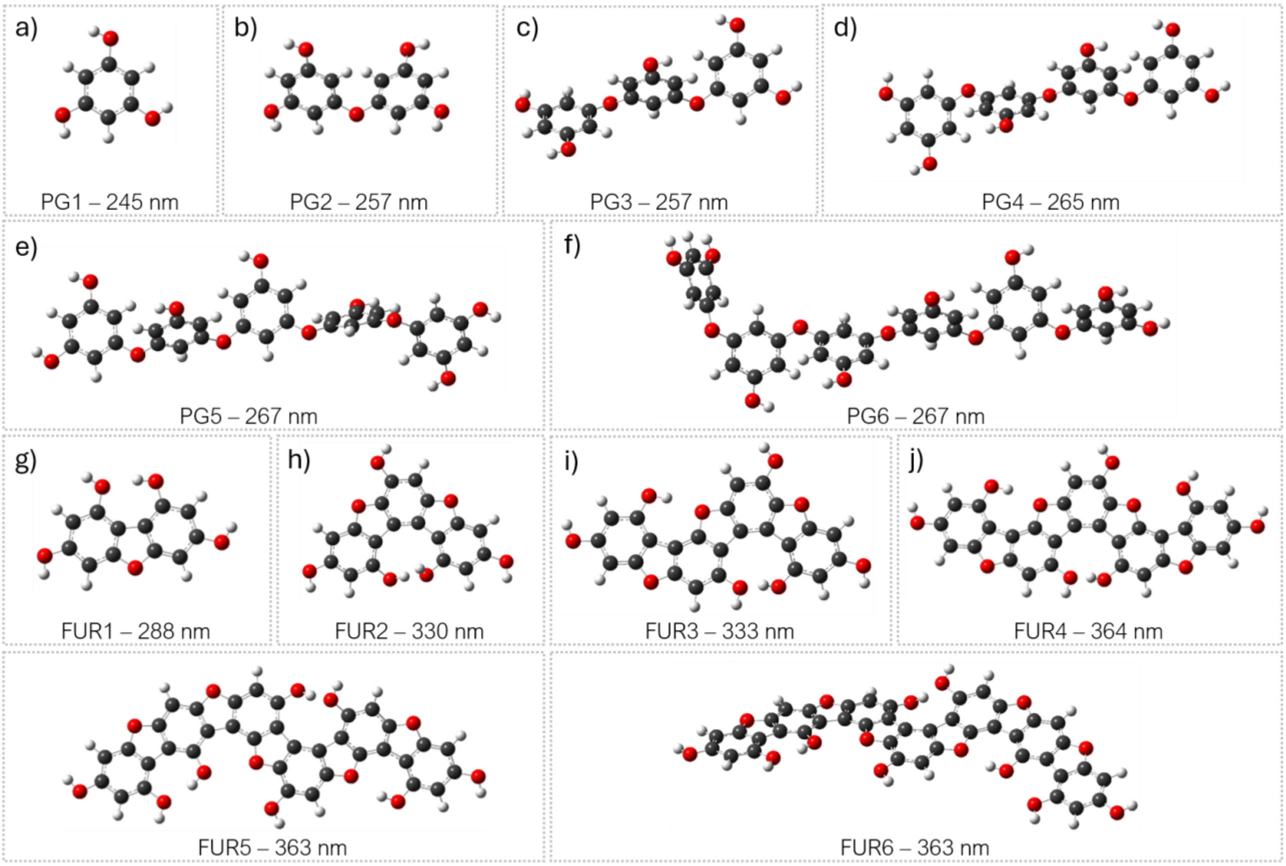

Figure S5. Optimized molecular structures and calculated HOMO–LUMO gaps of the investigated phloroglucinol-derived oligomeric models. (a–f) PG-polymer structures with increasing number of repeating units, from PG1 to PG6. (g–l) Furan-based structures with increasing number of units, from FUR1 to FUR6. The corresponding HOMO–LUMO gap values are reported below each structure.

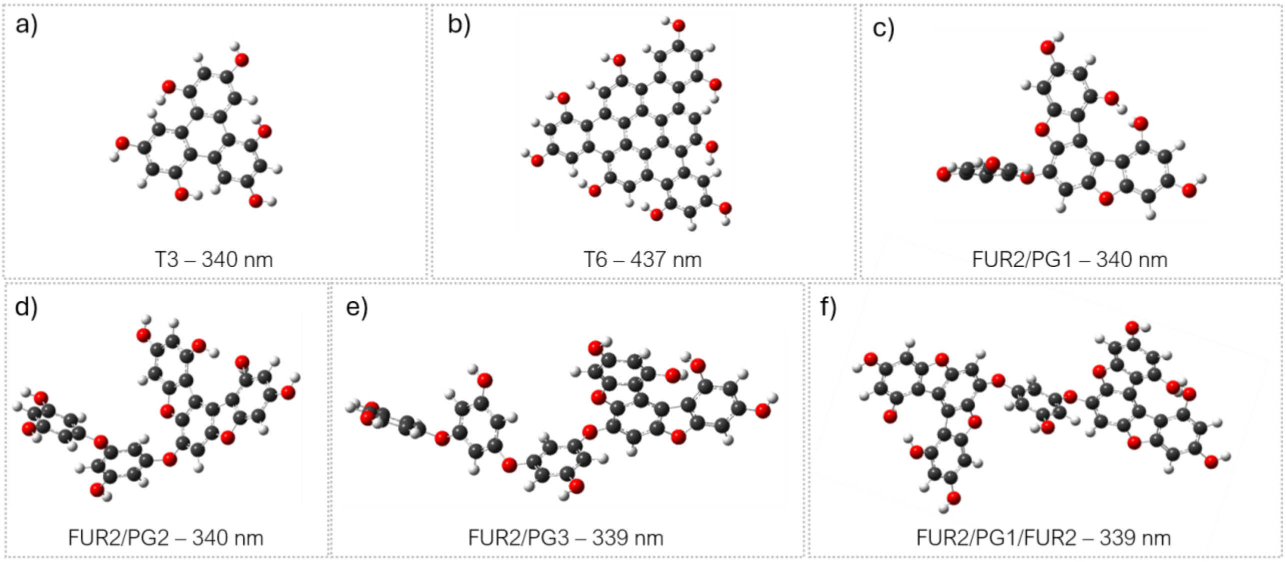

Figure S6. Optimized molecular structures and calculated HOMO–LUMO gaps of additional phloroglucinol-derived structural models. (a,b) Trimeric and hexameric fused-ring models, T3 and T6. (c–f) Mixed furan/PG-

polymer models with different combinations of furan-based and PG units. The corresponding HOMO–LUMO gap values are reported below each structure.
